# YBX1 mediates alternative splicing and maternal mRNA decay during pre-implantation development

**DOI:** 10.1186/s13578-022-00743-4

**Published:** 2022-02-02

**Authors:** Mingtian Deng, Baobao Chen, Zifei Liu, Yongjie Wan, Dongxu Li, Yingnan Yang, Feng Wang

**Affiliations:** 1 Jiangsu Livestock Embryo Engineering Laboratory, College of Animal Science and Technology, Nanjing Agricultural University, Nanjing, 210095 China; 2 College of Animal Science and Technology, Nanjing Agricultural University, Nanjing, 210095 China

**Keywords:** Maternal to zygotic transition, YBX1, Alternative splicing, RNA decay

## Abstract

**Background:**

In mammals, maternal gene products decay and zygotic genome activation (ZGA) during maternal to zygotic transition (MZT) is critical for the early embryogenesis. Y-box binding protein YBX1 plays vital roles in RNA stabilization and transcriptional regulation, but its roles remain to be elucidated during pre-implantation development.

**Methods:**

In the present study, we re-analyzed transcriptional level of *YBX1* in mice, human, bovine, and goat embryos using public RNA-seq datasets. We further performed siRNA microinjection to knock down the expression of *YBX1*, and RNA sequencing of the 8-cell stage embryos in the control and *YBX1* knockdown group. To reveal the regulation mechanisms of YBX1, we conducted differentially expression analysis, alternative splicing (AS) analysis, enrichment analysis, and 5-EU staining using DESeq2, rMATs, clusterProfiler, and immunofluorescence technique, respectively.

**Results:**

The expression of *YBX1* was increased during MZT in goat, bovine, human, and mice, but significantly decreased in *YBX1* knockdown embryos compared with the controls, suggesting successfully knockdown of *YBX1*. The percentage of blastocyst was decreased, while embryos blocked at the 2- and 4-cell stage were increased in *YBX1* knockdown embryos compared to the controls. Using RNA-seq, we identified 1623 up-regulated and 3531 down-regulated genes in the 8-cell stage *YBX1* knockdown embryos. Of note, the down-regulated genes were enriched in regulation of RNA/mRNA stability and spliceosome, suggesting that YBX1 might medicate RNA stability and AS. To this end, we identified 3284 differential AS events and 1322 differentially expressed maternal mRNAs at the 8-cell stage *YBX1* knockdown embryos. Meanwhile, the splicing factors and mRNA decay-related genes showed aberrant expression, and the transcriptional activity during ZGA in goat and mice was compromised when *YBX1* was knocked down.

**Conclusion:**

YBX1 serves an important role in maternal mRNA decay, alternative splicing, and the transcriptional activity required for early embryogenesis, which will broaden the current understanding of YBX1 functions during the stochastic reprogramming events.

**Supplementary Information:**

The online version contains supplementary material available at 10.1186/s13578-022-00743-4.

## Background

After fertilization, maternal mRNAs were massively degraded through a critical developmental process known as maternal to zygotic transition (MZT), during which, the developmental control is handed from maternally provided gene products to those synthesized from the zygotic genome [1–4]. Recently, it was reported that additional maternal mRNAs degradation was also depend on zygotic genome activation (ZGA) in mice (Z-decay) [5]. Since maternal mRNAs degradation is the first step of developmental transitions after fertilization, it is of importance to investigate the mechanisms of mRNA decay during the pre-implantation development.

The maternal mRNAs degradation is sophisticated. Typically, general mRNA decay is initiated by deadenylation and mRNA decapping, through the CCR4-NOT (CNOT) deadenylase [6] and the decapping enzyme DCP1/2 [7], respectively. Recent studies revealed that epigenetic modifications and maternal factors play critical roles during mRNA decay. For example, B-cell Translocation Gene-4 (BTG4) bridged CNOT to EIF4E, and facilitated decay of maternal mRNA [8]. Deficiency of N6-methyladenosine (m6A) reader protein YTH N6-methyladenosine RNA binding protein 2 (YTHDF2) decelerated the decay of m6A-modified maternal mRNAs and impeded ZGA initiation [9, 10]. These and subsequent studies suggesting that maternal factors play pivotal roles during the pre-implantation development [5, 11].

Y-box binding proteins, including YBX1, were discovered to bind to Y-box DNA elements, and are expressed in bacteria and animals [12]. It was reported that Y-box binding proteins are enriched in oocytes [13], and were identified as major components of cytoplasmic messenger ribonucleoproteins (mRNPs), with ubiquitous RNA-binding ability [14]. They were linked to a wide range of nucleic acid-related processes including translational repression, RNA stabilization, and transcriptional regulation in cell culture systems [12]. YBX1 is required for cell proliferation in cancer cells [15]. Of note, Wang et al. reported that YBX1 was differentially expressed during follicular development [16], meanwhile, Violeta et al. reported that YBX1 is altered in pregnancy-associated disorders [17]. Moreover, Lu et al. and Uchiumi et al. found embryonic lethality in YBX1 deficiency mice at E13.5, due to multiorgan hypoplasia and abnormal patterns of cell proliferation within the neuroepithelium [18, 19]. These studies suggest essential roles of YBX1 during implantation development. However, it remains unclear whether YBX1 plays critical roles during the pre-implantation development.

Alternative splicing (AS) diversifies the repertoire of mature cellular mRNAs, and broadly shapes mRNA metabolism by exposing or eliminating binding sites for RNA-binding proteins or ncRNAs [20, 21]. It also influences mRNA stability and translational efficiency [22]. AS displayed tissue-specific distribution and developmental specificity [23, 24]. During oocyte meiotic maturation, AS was related to the regulation of transcription and mitochondrial translation [25]. Recently, Tian et al. and Cheng et al. found that AS also occurs during the early embryo development. They identified major wave of AS switches around MZT and they further reported relationship between AS and gene transcription during the process in mice [26, 27], suggesting that MZT might be in the tight control of AS regulatory networks. However, regulation of AS during the pre-implantation remains to be elucidated.

In the present study, we investigated the role of YBX1 and its regulatory mechanisms by knockdown experiments, RNA-seq, AS analysis, and 5-EU staining. We report that YBX1 serves an important role in maternal mRNA decay, alternative splicing, and transcriptional activity required for pre-implantation development. Our data will be helpful in understanding the dynamic regulation of the early embryogenesis.

## Results

### Up-regulation of *YBX1* during MZT in goat, bovine, mice, and human

**Fig. 1:**
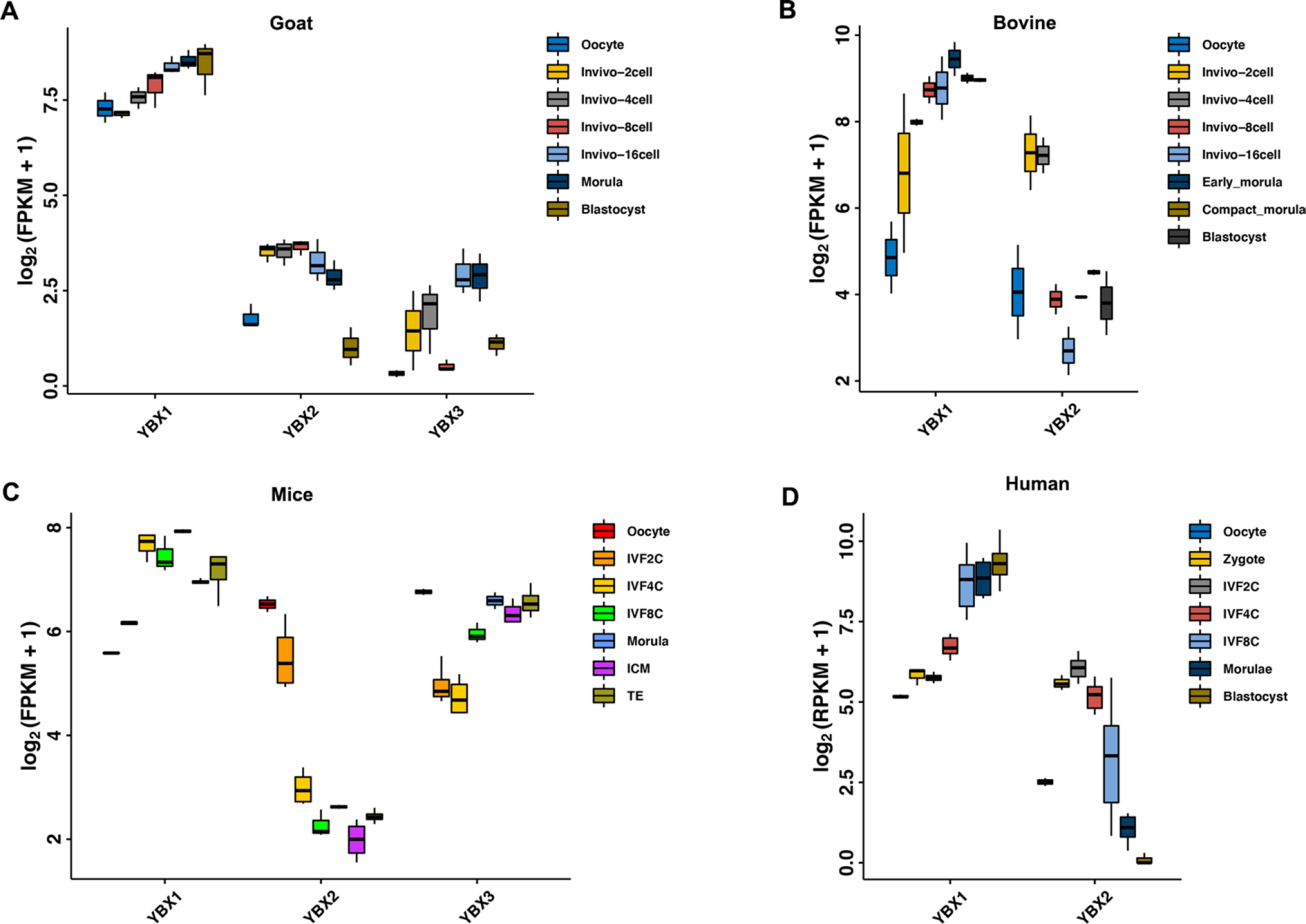
Up-regulation of *YBX1* during MZT in goat, bovine, mice, and human. **A** The expression of *YBX1*, *YBX2*, and *YBX3* in goat in vivo embryos. **B** The expression of *YBX1* and *YBX2* in bovine in vivo embryos. **C** The expression of *YBX1*, *YBX2*, and *YBX3* in in vitro fertilized embryos in mice. **D** The expression of *YBX1* and *YBX2* in in vitro fertilized embryos in human. FPKM/RPKM: fragments/reads per kilobase of exon model per million mapped fragments

We first investigated the expression pattern of *YBX* during the early embryo development in mice, human, and goat by re-analyzing public RNA-seq datasets. As shown in Fig. 1A, *YBX1* was gradually up-regulated from oocyte to blastocyst in goat; *YBX2* was up-regulated from the 2-cell embryos to the morula, while the expression of *YBX3* was deceased at the 8-cell stage embryos compared to the 4- and 16-cell stage embryos. During bovine early embryo development, the expression of *YBX1* was significantly increased in the 8-cell embryos compared to the 4-cell embryos, whereas *YBX2* was down-regulated during MZT (Fig. 1B). In mice, the expression of *YBX1* was increased after fertilization and further increased at the 4-cell embryos compared to the 2-cell embryo. *YBX2* and *YBX3* was down-regulated during ZGA in mice (Fig. 1C). The expression of *YBX1* in human were similar with that of mice (Fig. 1D). Conservative analysis revealed high homology of the protein sequence among goat, bovine, mice, and human (Additional file 1: Fig. S1). These data suggest conserved expression pattern of *YBX1* in mammalian embryos and that YBX1 might play vital roles during MZT.

### Knockdown of *YBX1* impeded the early embryo development

**Fig. 2:**
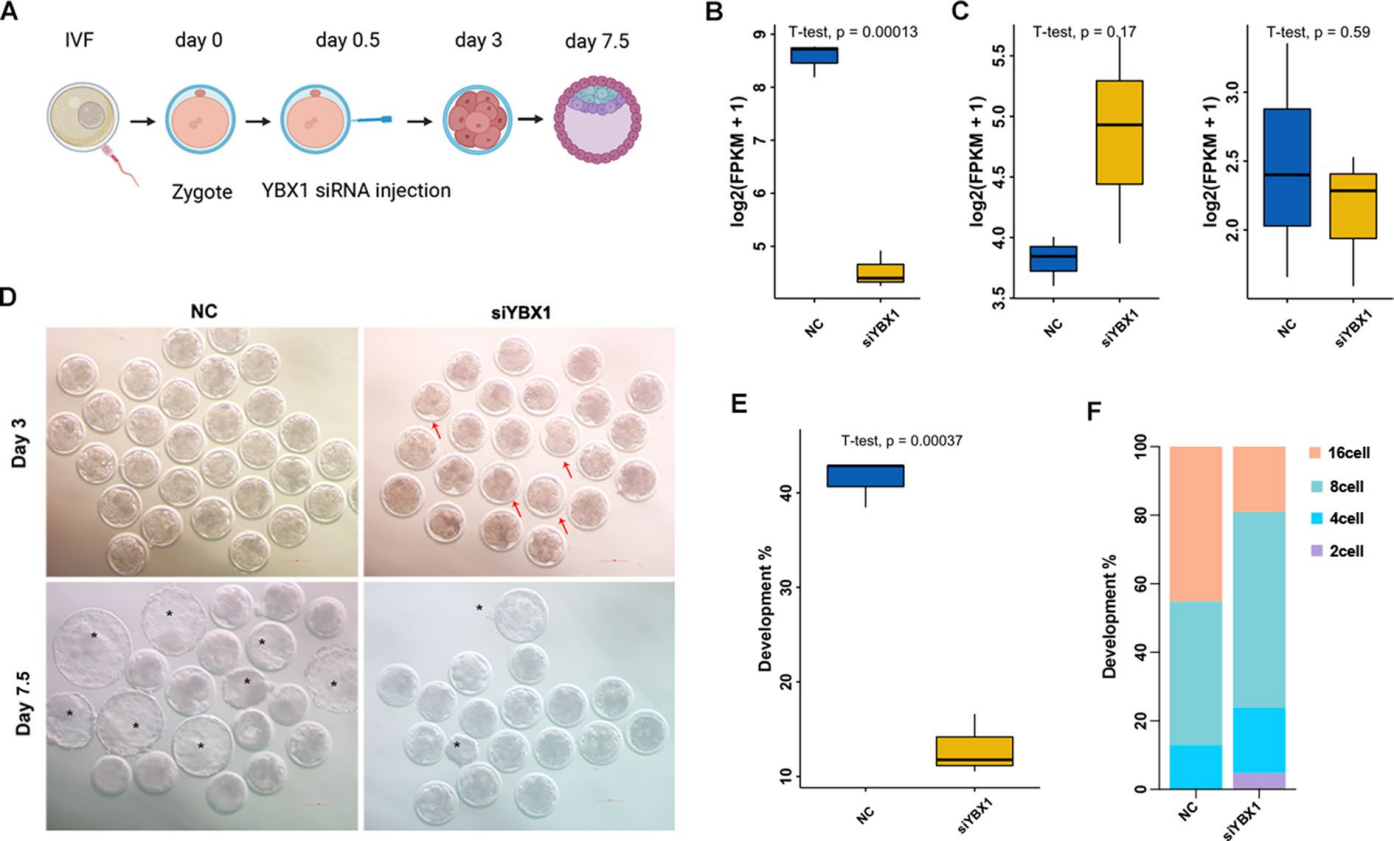
Knockdown of *YBX1* impeded the early embryo development in goat. **A** Schematic illustration of the knockdown experimental approach. **B** Characterization of *YBX1* expression at the 8-cell stage YBX1 knockdown embryos. **C** No statistical change of *YBX2* and *YBX3* at the 8-cell stage YBX1 knockdown embryos. **D** Representative images of *YBX1* knockdown and the control embryos at day 3 and day 7.5. The red arrow indicates developmental arrest embryos. The black asterisk indicates blastocysts. **E**, **F** Statistical analysis of embryos at day 7.5 and day 3 in *YBX1* knockdown embryos

To confirm its role in preimplantation, we knocked down *YBX1* by siRNA microinjection (Fig. 2A). The expression of *YBX1* was successfully inhibited, as revealed by RNA-seq and quantitative PCR (Fig. 2B and Additional file 1: Fig. S2), while the expression of *YBX2* and *YBX3* showed no statistical change in *YBX1* knockdown embryos at the 8-cell stage (Fig. 2C). Specifically, the percentage of blastocyst was significantly decreased in *YBX1* knockdown embryos compared to the controls (12.96 ± 1.51% vs. 41.39 ± 1.20%, p < 0.01; Fig. 2D, E). Moreover, embryos that blocked at the 2- and 4-cell stage were increased in *YBX1* knockdown embryos compared to the controls (Fig. 2F), suggesting essential roles of YBX1 during the early embryo development.

### Knockdown of *YBX1* compromised the transcriptional activity during ZGA

**Fig. 3:**
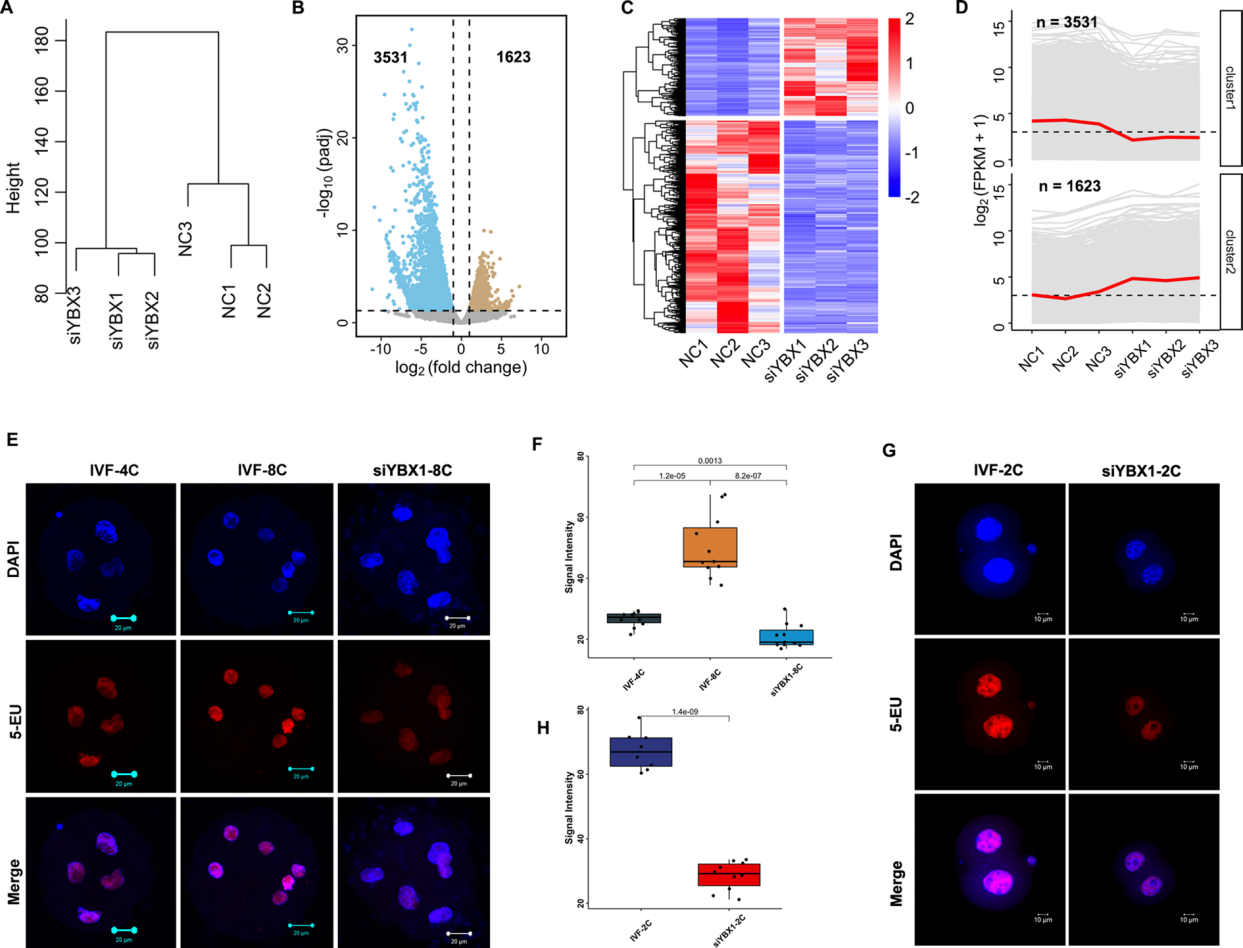
Knockdown of *YBX1* Compromised the transcriptional activity during ZGA. **A** Unsupervised clustering of genes in *YBX1* knockdown and the control embryos. **B** Volcano plot of gene expression after *YBX1* knockdown. **C** Heatmap of DEGs at the 8-cell stage *YBX1* knockdown embryos and the controls. **D** Two clusters of the DEGs. **E** Representative images 5-ethynyl uridine (5-EU, red) and DAPI staining (blue) at the 4- and 8-cell embryos, and the 8-cell stage *YBX1* knockdown embryos in goat. Scale bar = 20 μm. **F** Statistical analysis of 5-EU signal intensity in 4- and 8-cell embryos, and the 8-cell stage *YBX1* knockdown embryos in goat. Values are presented as mean ± SEM and compared using student’s t-test. **G** Representative images 5-EU (red) and DAPI staining (blue) at the 2-cell stage *YBX1* knockdown embryos and the controls in mice. Scale bar = 10 μm. **H** Statistical analysis of 5-EU signal intensity in 2-cell stage *YBX1* knockdown embryos and the controls in mice. Values are presented as mean ± SEM and compared using student’s t-test

To determine whether gene expression profiles correlated with the treatment, we analyzed RNA-Seq data by unsupervised hierarchical clustering. Embryos that clustered together were at the same group (Fig. 3A), and the fragments per kilobase of exon model per million mapped fragments (FPKM) value showed no significant change between YBX1 knockdown embryos and the controls (Additional file 1: Fig. S3), indicating good quality of RNA-seq data. Using DESeq2, we obtained 5154 differentially expressed genes (DEGs). Of which, 3531 genes were down-regulated, while 1623 genes were up-regulated at the 8-cell stage *YBX1* knockdown embryos compared to the controls (Fig. 3B-D, Additional file 2: Table S1), suggesting compromised transcriptional activity during ZGA by *YBX1* knockdown. We further performed 5-EU staining to confirm the notion. As expected, 5-EU was weakly stained in the 4-cell embryos, and markedly increased in the 8-cell embryos. However, in the 8-cell stage *YBX1* knockdown embryos, the level of 5-EU was decreased compared to the 4- and 8-cell embryos in the control group (p < 0.01, p < 0.001; Fig. 3E, F). In mice, ZGA initiates at the 2-cell embryos. The 5-EU was strongly stained at the 2-cell embryos, while the signal intensity of 5-EU was significantly decreased at 2-cell YBX1 knockdown embryos in mice (p < 0.001; Fig. 3G, H). These data suggest that knockdown of *YBX1* impaired transcriptional activity during ZGA in both goat and mice.

**Fig. 4:**
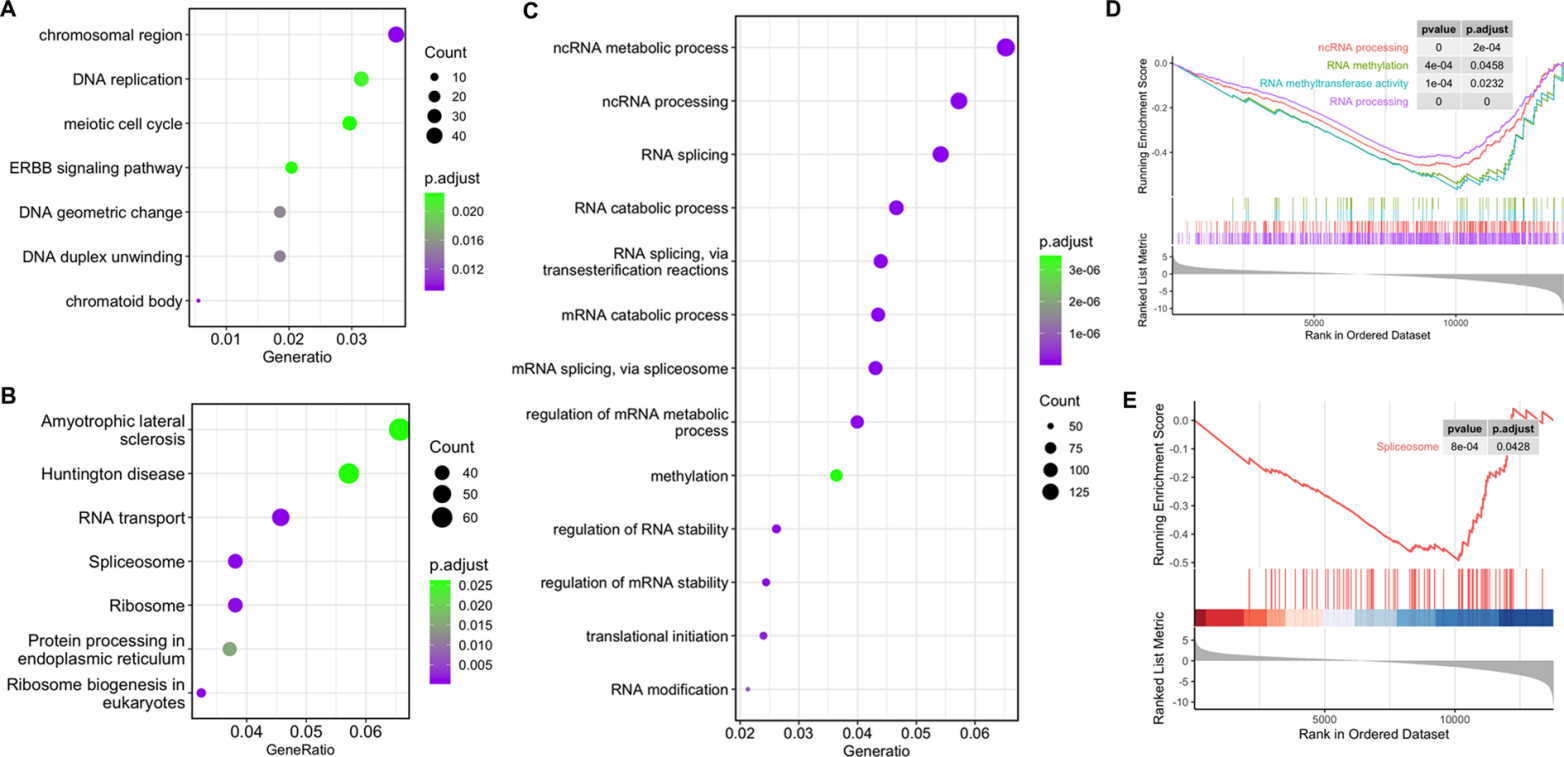
Enrichment analysis of DEGs in YBX1 knockdown embryos. GO enrichment of up-regulated **A** and downregulated **B** DEGs. **C** KEGG enrichment of all DEGs. **D** Enriched GO items revealed by GSEA. **E** Enriched KEGG items revealed by GSEA. *DEGs* differentially expressed genes

It is important to know the pathway that genes were de-repressed and/or downregulated in knockdown experiments. As shown in Fig. 4A, the 1623 up-regulated genes were enriched in chromosomal region, DNA replication, meiotic cell cycle, ERBB signaling pathway, DNA geometric change, and DNA duplex unwinding as revealed by GO annotation. The 3531 down-regulated genes were enriched in RNA/mRNA/ncRNA metabolic process, ncRNA processing, regulation of mRNA metabolic process, methylation, RNA modification, RNA/mRNA splicing, and regulation of RNA/mRNA stability (Fig. 4B). KEGG analysis revealed that the down-regulated genes were enriched in RNA transport, spliceosome, and ribosome (Fig. 4C). To further confirm GO and KEGG annotation results, we performed GSEA analysis. As expected, the DEGs were enriched in ncRNA processing (p.adjust < 0.001), RNA methylation (p.adjust < 0.05), RNA methyltransferase activity (p.adjust < 0.05), RNA processing (p.adjust < 0.001), and spliceosome (p.adjust < 0.05; Fig. 3D, E). These data suggest that YBX1 regulate MZT by regulation of RNA splicing and RNA stability.

### *YBX1* regulates alternative splicing during MZT

**Fig. 5:**
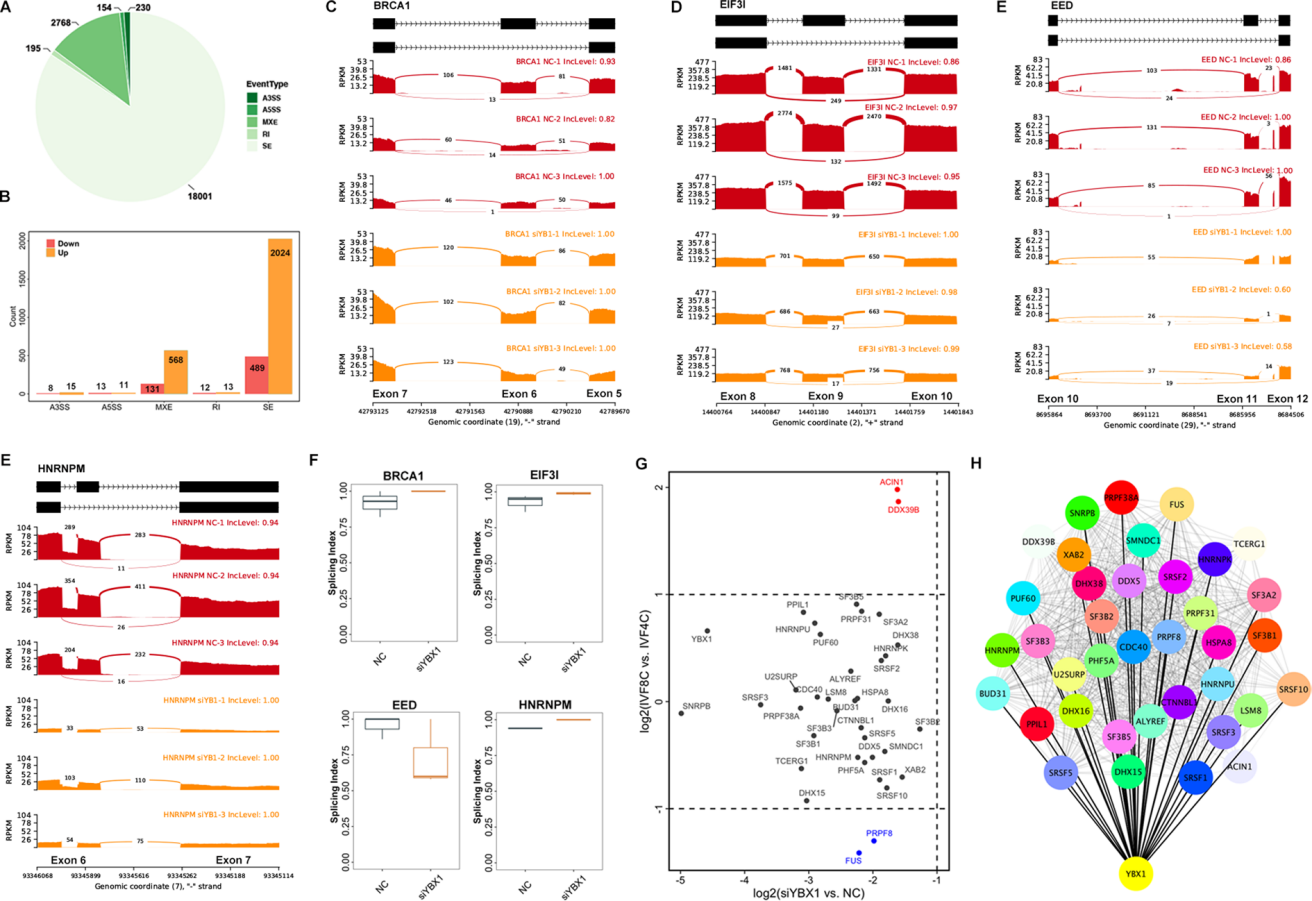
*YBX1* regulates alternative splicing during MZT. **A** Pie chart of alternative splicing events identified in the 8-cell stage *YBX1* knockdown embryos and the controls.** B** Differential alternative splicing after *YBX1* knockdown. **C**–**E** Exon skipping in the sixth exon of BRCA1, the ninth exon of EIF3I, the eleventh exon of EED, and the seventh exon of HNRNPM. **F** Boxplot of splicing index of the four genes. **G** Expression scatterplot showing down-regulation of splicing factors. **H** High correlation of *YBX1* with the splicing factors

Since the DEGs were enriched in spliceosome, we further investigated the AS events in the 8-cell stage *YBX1* knockdown embryos by analyzing the RNA-seq data. As shown in Fig. 5A, 18,001 skipped exon (SE), 195 retained intron (RI), 2768 mutually exclusive exons (MXE), 154 alternative 5`splice site (A5SS), and 230 alternative 3`splice site (A3SS) was identified at the 8-cell embryos. With the △PSI > 0.05 and the false discovery rate (FDR) < 0.05, we identified 3284 differential AS events. SE (76.52%), followed by MXE (21.29%) appears to be the most abundant differential AS events (Fig. 5B, Additional file 3: Table S2). For example, knockdown of *YBX1* promoted the sixth exon skipping in Breast Cancer Type 1 (BRCA1, Fig. 5C), but inhibited the ninth exon skipping in Eukaryotic Translation Initiation Factor 3 Subunit I (EIF3I, Fig. 5D), the eleven-exon skipping in Embryonic Ectoderm Development (EED, Fig. 5E), and the sixth exon skipping in Heterogeneous Nuclear Ribonucleoprotein M (HNRNPM, Fig. 5F).

Transcriptional profile of splicing factors and genes of spliceosome pathway was further characterized at the 8-cell stage *YBX1* knockdown embryos. During ZGA, Serine and Arginine Rich Splicing Factor (SRSF)1/2/3/10, Splicing Factor 3b Subunit (SF3B) 1/3/5, HNRNPM, HNRNPK, HNRNPU, DExD-Box Helicase 39B (DDX39B) showed no statistically changed. However, they were down-regulated in the 8-cell stage *YBX1* knockdown embryos compared to the controls (Fig. 5G). In addition, these genes were not only highly correlated with each other, but also predicted to be targeted with the YBX1 (Fig. 5H). Taken together, our data suggest that YBX1 was in regulation of AS during the MZT process.

### Knockdown of *YBX1* impairs maternal mRNA decay during MZT

**Fig. 6:**
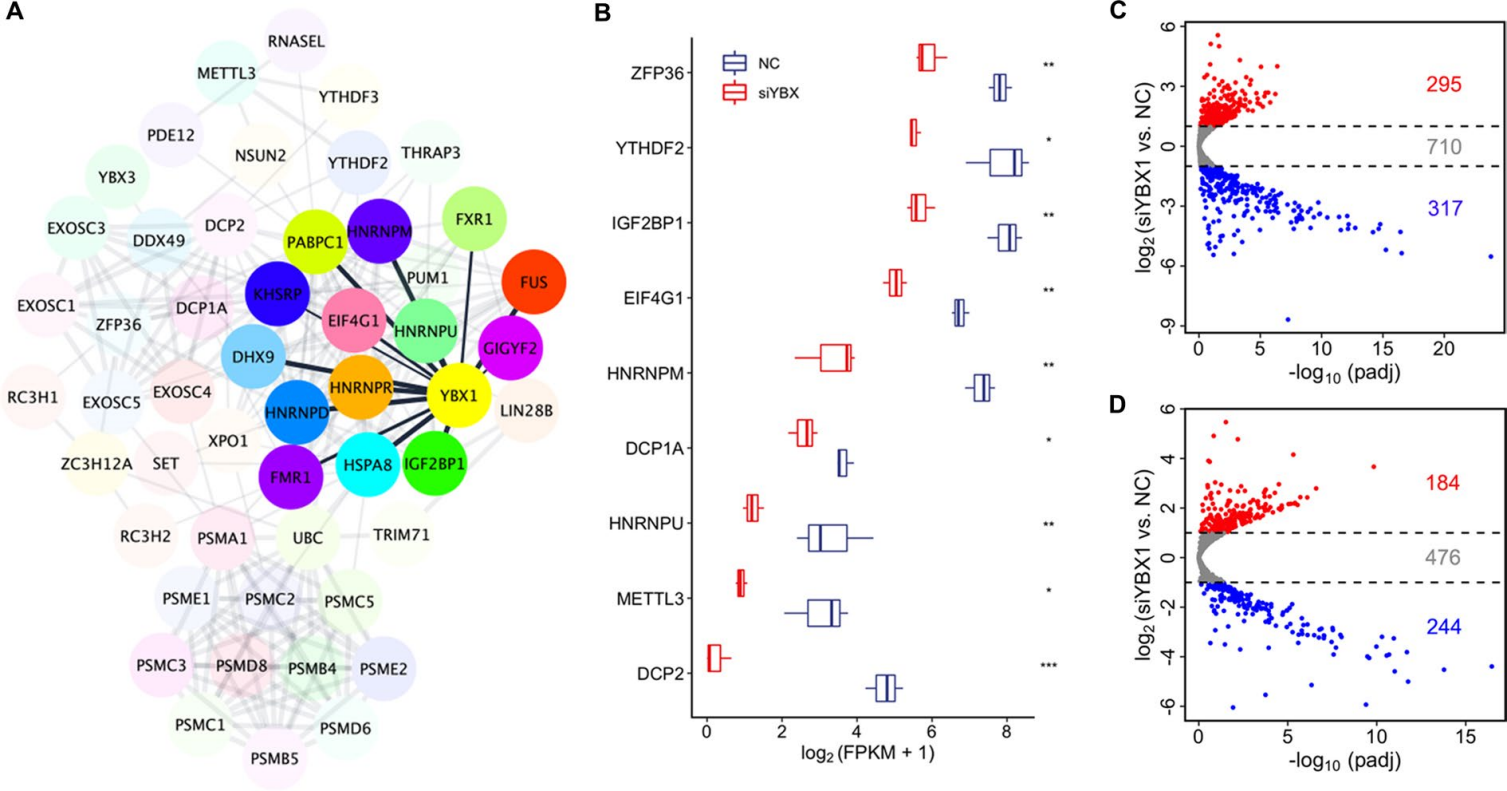
Knockdown of *YBX1* impairs maternal mRNA decay during MZT. **A** High correlation of *YBX1* with the mRNA stability related genes. **B** Boxplot showing down-regulation of critical mRNA stability related genes. **C** Volcano plot of M-decay maternal mRNAs at the 8-cell stage *YBX1* knockdown embryos. **D** Volcano plot of Z-decay maternal mRNAs at the 8-cell stage *YBX1* knockdown embryos

To confirm YBX1 is associated with RNA stability, we established a highly correlated hub genes network. As shown in Fig. 6A, the N6-methyladenosine-related genes (YTHDF2/3, METTL3, and IGF2BP1), Proteasome (Prosome, Macropain) 26S Subunit (PSM) family, Heterogeneous Nuclear Ribonucleoprotein (HNRNP), Decapping mRNA 1A (DCP1A), and DCP2 were highly correlated. Moreover, EIF4G1, HNRNPD, HNRNPM, HNRNPR, HNRNPU, and IGF2BP1 were predicted to target with YBX1 directly (Fig. 6A). The expression of *ZFP36* (p < 0.01), *YTHDF2* (p < 0.05), *IGF2BP1* (p < 0.01), *EIF4G1* (p < 0.01), *HNRNPM* (p < 0.01), *HNRNPU* (p < 0.01), *DCP1A* (p < 0.05), *DCP2* (p < 0.001), and *METTL3* (p < 0.05) were significantly decreased in *YBX1* knockdown embryos at the 8-cell stage compared to the controls (Fig. 6B), suggesting that YBX1 might regulate mRNA decay during MZT.

We further analyzed the expression of maternal mRNAs in *YBX1* knockdown embryos. There were 1322 M-decay genes expressed in both the knockdown and the NC group. Of which, 295 maternal genes were up-regulated, while 317 maternal genes were down-regulated in *YBX1* knockdown embryos at the 8-cell stage compared to the controls (Fig. 6C). As for the Z-decay genes, 904 genes were detected in the RNA-seq data. In the 8-cell stage *YBX1* knockdown embryos, the expression of 184 Z-decay genes was increased, while the expression of 244 Z-decay genes was decreased compared to the controls (Fig. 5D). Taken together, these data suggested that knockdown of *YBX1* impaired the maternal mRNA decay.

## Discussion

YBX1 serves an important role in translation, RNA stabilization, and transcriptional regulation in cell culture systems. In the present study, we found knockdown of *YBX1* impede the pre-implantation in goat. We further reported that YBX1 regulates alternative splicing, maternal mRNA decay, and transcriptional activity during MZT. Consistent with the previous studies that YBX1 is enriched in oocytes of Xenopus and primate [13, 16], *YBX1* were highly expressed in the mature oocytes in goat, bovine, mice, and human, with the FPKM > 5, in the present study. Moreover, the expression of *YBX1* was further increased after fertilization, specifically during the MZT process, which is in line with the data of Georgia et al. [28]. Thereby, YBX1 might play conserved roles during the early embryo development. To this end, we found high homology of the protein sequence of YBX1 among goat, bovine, mice, and human.

We show that YBX1 is related to the maternal mRNA degradation. Supporting this finding, YBX1 in transcription regulation has been reported in cell culture system [29]. Consistently, the dysregulation of several mRNA decay related genes was found in the 8-cell stage YBX1 knockdown embryos. mRNA decapping is the ultimate step before rapid clearance of the mRNA and the decapping reaction is catalyzed by a multi-protein complex formed by the DCP2 catalytic subunit and its DCP1 cofactor [7]. Therefore, the down-regulation might impair the mRNA decay process. mRNA stability was also regulated by m6A. The m6A reader IGF2BP1 and YTHDF2 have been reported to regulate the early embryo development [9, 10, 30], while the m6A methyltransferase METTL3 was essential for fertility [31]. The down-regulation of *METTL3*, *IGF2BP1*, and *YTHDF2* might impede the early embryo development. Taken together, YBX1 might affect the maternal mRNA degradation through m6A. Indeed, a recent study reported that YBX1 is required for maintaining myeloid leukemia cell survival by regulating BCL2 stability in an m6A-dependent manner [15]. It will be of interest to investigate the correlation of YBX1 and m6A during the pre-implantation development. The transcriptional activity was inhibited when YBX1 was knocked down, which could be explained by the fact that ZGA occurs after maternal mRNA degradation, the impaired mRNA decay might lead to the transcriptional activity failure.

In addition, YBX1 represses global translation during oocyte maturation and the MZT process in zebrafish [14]. Consistent with the data, we found that targeting YBX1 impairs the goat MZT in vitro. We could not determine the dynamic changes of the translation due to the limited embryo samples. Nevertheless, using bioinformatic analysis, YBX1 was predicted to cross-talk with EIF4G1, which was down-regulated at the 8-cell stage YBX1 knockdown embryos. Previous studies revealed that EIF4G is the major scaffolding protein in the translation initiation complex [32], and YBX1 displaces EIF4G from capped and uncapped transcripts through its C terminal domain [29]. Therefore, YBX1 might target the EIF4G1 to medicate the translation during the MZT in goat.

AS plays a critical role in the regulation of gene expression and protein diversity in a variety of eukaryotes. Previous studies revealed that major wave of AS switches around MZT and there was relationship between the AS events and the gene transcription during MZT in mice [26, 27]. Consistent with these data, we identified 21,348 AS events and 3284 differential AS events in the present study. Specifically, AS events occur in exons of BRCA1 and EED. Consistently, AS of these genes have been reported in cancer cells [33, 34]. Given that EED and BRCA1 is required for the early embryo development [35, 36], the differential AS in exons of BRCA1 and EED might lead to abnormal development of pre-implantation. In addition, we found that YBX1 medicates the AS events in the early embryo development. Supporting this finding, Jayavelu et al. reported that knockdown of YBX1 promotes RI events in mouse JAK2VF cells [37]. In the present study, targeting of YBX1 promotes SE and MXE events at the 8-cell stage *YBX1* knockdown embryos. Thereby YBX1 might medicate the pre-implantation development by regulation of AS in exons of critical genes.

## Conclusions

Our results identify that YBX1 is essential for the early embryo development. We further reported that YBX1 serves an important role in maternal mRNA degradation, alternative splicing, and the transcriptional activity. These data will advance the current understanding of YBX1 functions during the MZT process.

## Methods

### In vitro fertilization

In vitro maturation (IVM) was performed as previously described [38]. Briefly, cumulus-oocyte complexes (COCs) with more than two layers of compact cumulus cells and a dense, homogeneous cytoplasm were obtained and cultured in groups of 20 in 60-μL droplets of IVM medium at 38.5 °C, with 5% CO_2_, 95% air, and saturated humidity, for 22–24 h. Subsequently, in vitro fertilization (IVF) was performed as previously described [10]. After culturing for 16 h at 38.5 °C, with 5% CO_2_, 5% O_2_, 90% N_2_, and saturated humidity, the zygotes were collected for knockdown experiment. Mice IVF were performed as described in our previous study [39].

### Expression of YBX family during goat, bovine, human, and mice MZT

RNA sequencing (RNA-seq) data of goat (PRJNA543590), bovine (GSE59186), human (GSE36552), and mice (GSE98150) were downloaded from Gene Expression Omnibus. Gene expression was normalized with fragments/reads per kilobase of exon model per million mapped fragments (FPKM/RPKM).

### Knockdown of *YBX1*

As goat *YBX1* shared high homology with that of bovine, human, and mice (Additional file 1: Fig. S1), we obtained the sequence of small interfering RNA (siRNA) against YBX1 from a previous study [40], and synthesized at GenePharma (Shanghai, China). In general, 5–10 pL of 20 µM siRNAs targeted *YBX1* were microinjected into the cytoplasm of zygotes. The MISSION siRNA universal negative control was served as a negative control (NC) for knockdown experiment. Goat microinjected zygotes were cultured as described in our previous study [41]. Development status was determined at 72 and 168 h after microinjection. Embryos were collected at 72 h after microinjection for RNA-seq.

### Gene expression analysis

Gene expression analysis was performed as described in our previous study [10]. Briefly, cDNA was synthesized using cellAmp whole transcriptome amplification kit (Takara, Dalian, China) following the manufacturer’s instruction, and quantitative PCR was performed on an ABI 7300 Real-Time PCR System. Relative mRNA expression was normalized to *Gapdh* and calculated using the 2^−ΔΔCt^ method. Primers are shown in Additional file 4: Table S3.

### RNA library construction and sequencing

To profile RNA expression in the 8-cell stage *YBX1* knockdown embryos and the controls, 30 of each (in 3 replicates) were pooled and directly lysed. RNA libraries were constructed using the Smart-seq2 method. Then, cDNA was fragmented by dsDNA fragmentase (M0348S, NEB) by incubating at 37 °C for 30 min, and size selection was performed with provided sample purification beads, then the fragmented cDNA at the size of 150–300 bp was used for library construction. Followed by paired-end sequencing on an illumina Novaseq 6000 platform (LC bio, Hangzhou, China).

### Read alignment and differential expression analysis

Quality control was performed to remove adaptors and low-quality bases using fastp (v0.19.6). All reads that passed quality control were mapped to goat genome ARS1 using HISAT2 (v2.2.1) with default settings. Uniquely mapped reads were subsequently assembled into transcripts guided by the reference annotation using featureCounts (v2.0.1). Differential expression analysis was performed using DESeq2 (v3.11). Genes with log 2 (fold change) > 1 or < -1 and with p-value < 0.05 were deemed as DEGs.

### GO, KEGG, and GSEA analysis

Gene Ontology (GO) annotation and Kyoto Encyclopedia of Genes and Genomes (KEGG) pathway enrichment analyses of upregulated and downregulated DEGs were conducted separately using clusterProfiler R package (v3.12.0). GO and KEGG terms with an FDR adjust p-value < 0.05 were deemed statistically significant. Gene Set Enrichment Analysis (GSEA) of all DEGs were performed with clusterProfiler and enrichplot R package (v1.10.2). The minimal and maximal size of each geneSet and pvalue cutoff were set to 10, 1000, and 0.05, respectively.

### Series test of cluster analysis

To classify the stage-specific gene expression, we performed k-means clustering on DEGs in *YBX1* knockdown and the control embryos (k = 2) as described in our previous study [42].

### 5-EU incorporation assay

Newly synthesized mRNA was detected as previously described [38]. Briefly, embryos were placed in BO-IVC medium containing 2 mM 5-EU for 2 h at 38.5 °C, followed by fixing in 4% paraformaldehyde for 30 min, permeabilizing in 0.5% Triton X-100 for 10 min, incubation with Apollo reaction cocktail for 30 min, and permeabilizing in 0.5% Triton X-100 for another 10 min at room temperature. After staining with DAPI for 3 min, the embryos were mounted on glass slides with a drop of antifade mounting medium (Beyotime, Beijing, China). Imaging was obtained using LSM710 laser scanning confocal microscope (Carl Zeiss, Oberkochen, Germany), and signal intensity was assessed with ImageJ software (v1.52a).

### Differential alternative splicing identification

The improvement of single cell RNA-seq technology and computational analysis have facilitated comprehensive analysis of AS in single cells. Using the bam files generated in RNA-seq data analysis, we identify the different types of AS events using the rMATS (v4.1.1). Cutoffs of FDR and inclusion level difference [△percent spliced index (PSI)] at 0.05 were used to screen for statistically significant differential AS. The AS genes were visualized with rmats2sashimiplot (v2.0.4).

### Data visualization and statistical analysis

R programming language was mainly used in statistical analysis (student’s t test) and data visualization. Heatmap and boxplot of volcano, gene expression, GO, KEGG, and signal intensity were generated using R package pheatmap (v1.0.12) and ggplot2 (v3.3.2), respectively. Clustering analysis was performed using hclust, using average method.

## Supplementary Information

**Additional file 1: Figure S1. **High homology of YBX1 protein sequence among goat, bovine, mice, and human. **Figure S2.** The expression of YBX1 was successfully knocked down at the 8-cell stage embryos compared to the controls, as revealed by quantitative PCR. Student’s t test, **p < 0.01. **Figure S3.** Boxplot revealed no significant change of all genes’ FPKM between YBX1 knockdown embryos and the controls.

**Additional files 2: Table S1. **Differentially expressed genes at the 8-cell stage YBX1 knockdown embryos.

**Additional files 3: Table S2. **Differential alternative splicing events between the 8-cell stage YBX1 knockdown embryos and the controls.

**Additional file 4. Table S3. **Details of primer sequences, expected product size, and annealing temperature (Tm, °C) of genes used for quantitative PCR.

## Data Availability

The accession number of the RNA-seq data reported in this paper is GEO: GSE182908.
