# Supplementary material for: YBX1 mediates alternative splicing and maternal mRNA decay during pre-implantation development
Source: Cell Biosci. 2022 Feb 2;12:12. doi: 10.1186/s13578-022-00743-4 (PMC8812265; doi:10.1186/s13578-022-00743-4)
Supplement: Supplementary file 1 — Additional file 1: Figure S1. High homology of YBX1 protein sequence among goat, bovine, mice, and human. Figure S2. The expression of YBX1 was successfully knocked down at the 8-cell stage embryos compared to the controls, as revealed by quantitative PCR. Student’s t test, **p < 0.01. Figure S3. Boxplot revealed no significant change of all genes’ FPKM between YBX1 knockdown embryos and the controls. [file 13578_2022_743_MOESM1_ESM.docx]

**
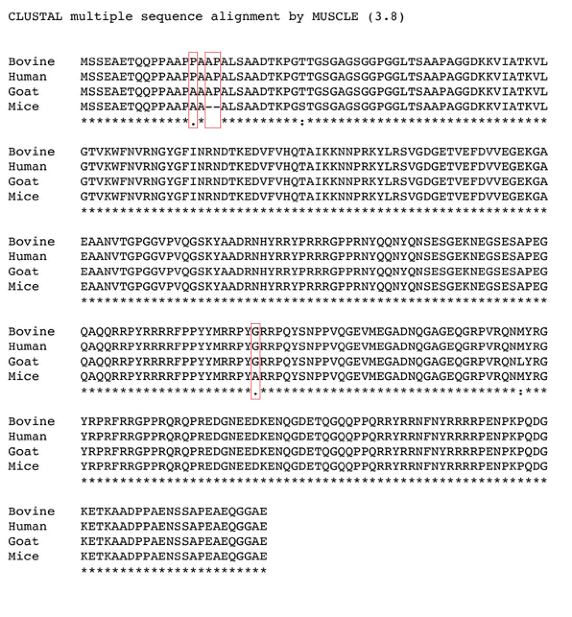
Additional file 1: Figure S1.** High homology of YBX1 protein sequence among goat, bovine, mice, and human.


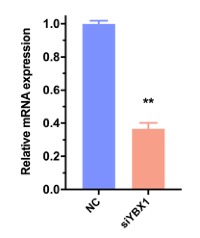
 **Additional file 1: Figure S2.** The expression of YBX1 was successfully knocked down at the 8-cell stage embryos compared to the controls, as revealed by quantitative PCR. Student’s t test, ** p < 0.01.

**Additional file 1: Figure S3** Boxplot revealed no significant change of all genes’ FPKM between YBX1 knockdown embryos and the controls.
