# Supplementary material for: YBX1 mediates alternative splicing and maternal mRNA decay during pre-implantation development
Source: Cell Biosci. 2022 Feb 2;12:12. doi: 10.1186/s13578-022-00743-4 (PMC8812265; doi:10.1186/s13578-022-00743-4)
Supplement: Supplementary file 4 — Additional file 4. Table S3. Details of primer sequences, expected product size, and annealing temperature (Tm, °C) of genes used for quantitative PCR. [file 13578_2022_743_MOESM4_ESM.docx]

**Additional file 4: Table S3. Details of primer sequences, expected product size, and annealing temperature (Tm, °C) of genes used for quantitative PCR.**

| *Target gene* | Primer sequence (5‘-3’) | Product size (bp) | Accession number | Tm (°C) |
| --- | --- | --- | --- | --- |
| *YBX1* | F-CCCCAGGAAGTACCTTCGC  R-AGCGTCTATAATGGTTACGGTCT | 158 | XM_018042673 | 59.4 |
| *Gapdh* | F-CGACTTCAACAGCGACACTCAC  R-CCCTGTTGCTGTAGCCCAATTC | 118 | NM_001034034 | 58.0 |
